# American Medical Association—Eleventh Annual Meeting

**Published:** 1858-06

**Authors:** 


### EXTRACTS.

### AMERICAN MEDICAL ASSOCIATION.—ELEVENTH ANNUAL MEETING.

### FIRST DAY.

The association met in the lecture-room of the Smithsonian Institution, and was called to order at a quarter past eleven o'clock a. m., by Dr. Condie, of Philadelphia, when the chair was taken by the president, Dr. Paul F. Eve, of Nashville, Tennessee. Vice Presidents Breckinridge of Kentucky, Reese of New York, and Campbell of Georgia, were also upon the platform; and at their table were the efficient secretaries, Drs. Foster of Tennessee, and Semmes of this city, to whom we are under obligations.

Rev. Byron Sunderland, D. D., at the invitation of the president, offered an eloquent and appropriate prayer, invoking the blessing of Almighty God upon the convention.

Dr. Harvey Lindsley, of this city, chairman of the committee of arrangements, then delivered an

### ADDRESS OF WELCOME.

The secretary then called the roll by States as it had been made out up to the commencement of the meeting, and the following number of delegates responded: Maine 2, New Hampshire 8, Connecticut 18, Vermont 1, Massachusetts 40, Rhode Island 5, New York 73, New Jersey 25, Pennsylvania 66, Delaware 4, Maryland 24, District of Columbia 25, Virginia 8, North Carolina 8, South Carolina 10, Georgia 12, Alabama 1, Kentucky 9, Tennessee 7, Indiana 6, Illinois 12, Michigan 3, Iowa 3, Missouri 4, Ohio 14, California 1, American Medical Society of Paris 1, U. S. Navy 2. [When the name of Dr. Harvey, who has come from California expressly to attend this convention, was called, there was loud applause.] Other members were announced at different times during the day, and when the association adjourned there were *four hundred and six* names registered. A large increase is expected to-day.

Dr. Lindsley, chairman of the committee of arrangements, reported that it had been decided to hold but one business session each day, from nine a. m. until three p. m. He also announced that the President of the United States would be happy to receive with those members of the association who might call at the executive mansion at eight o'clock in the evening such ladies as may accompany them. [Applause.]

On motion, the association confirmed the appointment of Dr. J. M. Snyder to fill a vacancy in the committee of arrangements.

On motion, it was decided that a nominating committee of one from each State represented should be raised, the delegation of each State selecting its representative therein. A brief discussion upon the propriety of permitting the army and navy delegations to appoint separate members of this committee was decided by the president in favor of their having the privilege, and the decision was sustained by the association.

There was then a recess of fifteen minutes, during which the different delegations assembled in various parts of the lecture-room to choose their representatives in the committee. After the meeting was called to order, the secretary read the list as follows:

*Committee of Nomination.*—Job Holmes, Maine; George H. Hubbard, New Hampshire; P. Pineo, Vermont; Ebenezer Alden, Massachusetts; Ashbel Woodward, Connecticut; J. Mauran, Rhode Island; H. D. Bulkley, New York; J. P. Colman, New Jersey; Isaac Hays, Pennsylvania; H. F. Askew, Delaware; S. P. Smith, Maryland; Noble Young, District of Columbia; A. S. Payne, Virginia; W. H. McKee, North Carolina; Wm. T. Wragg, South Carolina; Joseph P. Logan, Georgia; J. T. Hargraves, Alabama; R. J. Breckinridge, Kentucky; J. Berrian Lindsley, Tennessee; Wm. M. McPheeters, Missouri; George Mendenhall, Ohio; Calvin West, Indiana; A. H. Luce, Illinois; Zina Pitcher, Michigan; Thomas O. Edwards, Iowa; O. Harvey, California; and George Clymer, United States navy.

On motion, Drs. Bohrer of D. C., Flint of New York, and Hargraves of Alabama, were appointed by the president a committee on special essays.

Dr. David M. Reese, of New York, presented and read a written apology for having recommended for a position in Blockley Hospital, Philadelphia, Dr. McClintock, who had been expelled from the association for a violation of the ethics and the etiquette of the profession, by publishing a work on "domestic medicine," etc.

On motion of Dr. Condie, of Philadelphia, the apology was accepted, and ordered to be entered upon the minutes.

Dr. Bryan, of Philadelphia, who had also recommended Dr. McClintock, made a verbal adoption of Dr. Reese's apology, the reception of which was warmly debated. Dr. C. C. Cox, of Maryland, opposed, and Dr. Condie advocated the reception. Dr. A. B. Palmer, of Michigan, moved the previous question on a motion to refer the subject to a committee, which was lost. The apology of Dr. Bryan was then accepted.

Dr. Grafton Tyler, of Georgetown, D. C., chairman of the committee on prize essays, reported that the essays received were three in number, each of which had been examined with great care; considering, first, the intrinsic merits of each essay, and then their merits in relation to each other. The first prize was awarded to "an essay on the clinical study of the heart sounds, in health and disease," bearing the motto, "*Clinica clinice demonstrandum.*" The second prize was awarded to "an essay on vision and some of the anomalies as rendered by the ophthalmoscope," bearing the motto, "*Dux hominum medicus est.*"

Dr. Tyler then proceeded to open the sealed envelopes bearing the above-named mottoes, and containing the names of the writers of the essays. The first was written by Dr. Austin Flint, of Buffalo, New York; and the second by Dr. Montrose A. Pallen, of St. Louis, Missouri. This is the second time Dr. Flint has won this distinguished honor, and the third time that it has been awarded to Buffalo since the association was organized, eleven years ago.

On motion, the report of the committee was accepted and adopted. Doctors Flint and Pallen were then invited to give *resumes* of their essays, which they did.

Dr. Lindsley, from the committee of arrangements, then presented an invitation from Dr. Nichols to visit the Insane Asylum, and another from Rev. Mr. McGuire to visit Georgetown College.

On motion of Dr. Hamilton, of New York, these invitations were accepted, and the thanks of the association were returned therefor.

On motion of Dr. Lindsley, the Hon. Doctors Fitch, of Indiana, Chaffee, of Massachusetts, Clawson and Robbins, of New Jersey, and Shaw, of North Carolina, members of Congress, and Dr. Peter Parker, ex-commissioner to China, were elected "members by invitation," and requested to participate in the proceedings of the association.

On motion, Assistant Surgeon Frederick A. Rose, of the British navy, who so nobly volunteered his services on board the United States ship Susquehanna at Port Royal, and who came in her to New York, devoting himself to the sick crew, was unanimously elected a "member by invitation," and invited to take a seat upon the platform. [Applause.] It was announced that Dr. Rose had left the city.

Dr. Francis G. Smith, of Philadelphia, chairman of the committee on publication, made his report, showing the expense of publishing the annual volume.

Dr. Caspar Wistar, of Philadelphia, presented his annual report of receipts and expenditures, showing a balance on hand of $806. Accompanying the treasurer's report was a resolution providing that the back volumes on hand, when over two years old, shall be sold at two dollars a volume, and that volumes V, VII, VIII, and IX, of which there are a surplus, be sold at $5 a set to any member.

The special committee on medical education, of which Dr. G. W. Norris, of Philadelphia, is chairman, were called upon to report. There was no response; and, on motion, the subject was referred to the committee on nominations.

Dr. A. B. Palmer, chairman of the committee on medical literature, asked leave to defer his report until Wednesday, at 10 o'clock, which was granted.

A report was made by the committee on nominations, which was accepted, and the association then elected the following

OFFICERS.

*President*, Dr. Harvey Lindsley, of Washington city. *Vice Presidents*, Drs. W. L. Sutton, of Kentucky; Thomas O. Edwards, of Iowa; Josiah Crosby, of New Hampshire; and W. C. Warren, of North Carolina. *Secretary*, Dr. A. J. Semmes, of Washington city. [The other secretary will be elected when the location of the next association is selected.] *Treasurer*, Caspar Wistar, of Philadelphia.

On motion, Drs. Flint of New York, Gross of Pennsylvania, and Gibbes of South Carolina, were appointed a committee to conduct the president elect to the chair.

Dr. Lindsley, having been introduced to the association by the retiring president, Dr. Eve, made a few pertinent remarks, acknowledging the honor as the highest he had ever been called upon to receive, and the highest that any medical man in America can receive. [Applause.] Unaccustomed to preside over so large a body, and having had but little practice in presiding over smaller assemblages, he must throw himself upon the forbearance of the association and look to the members for support in the discharge of his official duties. [Applause.]

On motion, the thanks of the association were voted to the retiring officers for the able and impartial manner in which they have discharged the duties of their respective offices. [Applause.]

On motion, the ex-presidents of the association present were invited to take seats on the platform.

The committee on medical topography and epidemics was called by States. A paper from the member from Maine stated that he will report next year. There was no response from New Hampshire, Vermont, Rhode Island, Connecticut, or Massachusetts. Dr. Smith, of New Jersey, read an able report on New Jersey, and the association then adjourned until this morning at nine o'clock.

### EVENING HOSPITALITIES.

At eight o'clock in the evening, the delegates and the ladies who have accompanied them paid a visit by invitation to the Executive Mansion. The East Room, with the adjacent suite of drawing rooms, were brilliantly lighted, and were filled by about five hundred gentlemen, representing all sections of the country, and a hundred or more ladies. One of the delegates had seen upwards of fourscore years—others have but just entered upon the practice of their profession.

The President received his guests, as they were successively presented by Dr. Cornelius Boyle, chairman of the committee of arrangements, with his accustomed cordiality, and afterwards moved about in the East Room, engaged in conversation with the groups there gathered. The entire cabinet were present, with J. B. Henry, Esq., Marshal Selden, and Commissioner Blake.

From the Executive Mansion the delegates generally proceeded to Georgetown, where they were hospitably entertained at the residences of Dr. Grafton Tyler, at the corner of Gay and Washington streets, and of Dr. Riley, No. 91 Gay street. A cordial welcome and good cheer awaited them at the houses of each of these distinguished practitioners.

### SECOND DAY.

The association was called to order by the president, Dr. Harvey Lindsley, and A. J. Semmes, one of the secretaries, read the minutes of the first day's proceedings, which were adopted.

On motion of Dr. Watson, of New York, Dr. Delafield, of New York, one of the first officers of the association, was invited to take a seat on the platform.

On motion of Dr. Atkinson, of Virginia, an amendment to the constitution was received, providing that no person shall be recognized as a member or admitted as a delegate at meetings of the association who has been expelled from any State or local medical association until relieved by action of such State or local association.

Dr. Bond, of Maryland, asked to have the qualifications requisite for a seat read. He desired information as to the ethical qualifications for membership.

Dr. Watson, of New York, stated that, as by the constitution it was necessary to have amendments lie over one year, this was not a question for present debate.

The president decided that debate was not in order, and the amendment was accordingly laid on the table for consideration at the next annual meeting.

Dr. Boyle, chairman of the committee of arrangements, proposed the names of Doctors Huff and Knight, who were elected "members by invitation."

### REPORT ON MEDICAL LITERATURE.

Dr. A. B. Palmer, of Michigan, chairman of the committee on medical literature, made an able and interesting report.

On motion, the report was accepted, and ordered to be published.

On motion, Dr. Bozman, of Alabama, was elected a "member by invitation."

### REPORT ON MEDICAL EDUCATION.

Dr. James R. Wood, chairman of a special committee on medical education, made a lengthy report, discussing—1st, primary medical schools; 2d, the number of professorships in medical colleges; 3d, the length and number of terms during the year; 4th, the requisite qualifications for graduation; 5th, such other subjects of a general character as to give uniformity to our medical system. Having reviewed these propositions at length, the committee have arrived at the following conclusions:

*First.* Primary medical schools should be encouraged; but, as office instruction will continue to be sought by students, practitioners should either give them necessary advantages of demonstrations, illustrations, and recitations, or if not prepared to do so, they should refer them to such primary schools, or medical men, as will give them proper instruction.

*Second.* The number of professorships should not be less than seven, viz: a professor of Anatomy and Microscopy, Physiology and Pathology, Chemistry, Surgery, Practical Medicine, Obstetrics, and Materia Medica.

*Third.* There should be but one term annually, which should commence about the 1st of October, and close with the March following, thus lengthening the term to six months. The commencement of the term, in October, should be uniform in all the colleges throughout the country. During the session there should never be more than four lectures given daily.

*Fourth.* The qualifications for graduation, in addition to those now required by the schools, should be a liberal primary education, and attendance upon a course of clinical instruction in a regularly-organized hospital.

In order to give our medical colleges an opportunity to consider the recommendations here advanced, and that this body may have the advantage of their wisdom and their mature views, before any definite action is taken upon them, your committee submit to the association the following resolutions:

*Resolved,* That the several medical colleges of the United States be requested to send delegates to a convention to be held at on the day of for the purpose of devising a uniform system of medical education.

*Resolved,* That the present report of the special committee on medical education be referred to such convention for its consideration.

*Resolved,* That said convention of delegates from the several colleges of the United States be requested to submit to the meeting of this association in May, 1859, the result of their deliberations.

On motion, the report was accepted and referred to the committee on publication, the accompanying resolution being laid on the table.

The committee on nominations reported Louisville, Ky., as the place of meeting in 1859, and nominated Dr. S. S. Bemis, of that city, as second secretary. They also nominated the following standing committees:

*Committee on Publication*—Dr. F. Gurney Smith, Pa., chairman; Drs. Caspar Wistar, Pa.; A. J. Semmes, D. C.; S. M. Bemis, Ky.; S. L. Hollinsworth, Pa.; S. Lewis, Pa.; H. F. Askew, Del.

*Committee on Medical Literature*—Dr. John Watson, N. Y., chairman; Drs. L. A. Smith, N. J.; C. G. Comegys, Ohio; R. W. Gibbs, S. C.; W. M. McPheeters, Mo.

*Committee on Prize Essays*—Dr. J. B. Flint, Ky., chairman; Drs. M. Goldsmith, N. J.; H. Miller, Ky.; Calvin West, Ind.

*Committee on Medical Education*—Dr. G. W. Norris, Pa., chairman; Drs. A. H. Luce, Ill.; E. R. Henderson, S. C.; G. R. Grant, Tenn.; T. S. Powell, Ga.

*Committee of Arrangements*—R. J. Breckinridge, Ky., chairman; Drs. G. W. Ronald, B. M. Wible, D. W. Goodall, D. D. Thompson, N. B. Marshall, G. W. Burglass, R. C. Hewett, and A. B. Cook, all of Kentucky.

The report was accepted, the nominations were confirmed, and the committee received permission to sit again.

On motion of Dr. Hamilton, of Buffalo, the resolutions attached to the report of the committee on medical education were taken from the table.

Dr. Watson moved the appointment of a committee to consider the resolutions and report to-morrow [this] morning.

Dr. Bond thought that the subject had already been sufficiently discussed. It had been brought up year after year, occupying much of the time of the association, and he trusted that it would receive immediate consideration.

Dr. Davis, of Illinois, wished to have the subject made a special order for some time prior to the adjournment of the convention.

Dr. Rogers, of New York, wished to have the report printed, that all might have an opportunity of examining it and the propositions which it embodies.

Dr. Wood defended his report as a conservative report, just alike to the professors and to the laymen. He did not believe that any good could arise from a further discussion of the subject. None had arisen in years past—none could arise now. It was a bill of conciliation and of adjustment. Laymen of the profession merited censure for sending men to college not qualified for the profession, and colleges merited censure for sending men out not qualified to practice the healing art. [Applause.] He approved of the motion of Dr. Watson, that the report be submitted to a committee of delegates from colleges.

The report was referred to a select committee, to be composed of one member from each delegation representing a medical college or school.

On motion, thanks were voted to the late secretary, Dr. Foster, and his successor, Dr. Bemis, took his seat.

Dr. Hamm, of Pennsylvania, moved a suspension of the rules for the purpose of reconsidering the resolution of Dr. Condie, accepting the apology of Dr.Bryan. The vote upon suspending the rules stood—ayes 111, noes 82. The president ruled that a two-third vote was necessary, and decided the question as lost.

An appeal was taken from the decision of the chair, and the decision was not sustained. A vote was then taken, and the resolution accepting the apology of Dr. Bryan was reconsidered by a vote of—yeas 142, nays 70.

An attempt was then made to connect the resolution with that accepting the apology of Dr. Reese, but it was decided that it would first be necessary to dispose of the resolution reconsidered, and it was laid on the table.

A member from New Jersey hoped that the McClintock case would be brought fairly and squarely before the association, and that gentlemen would be made to "face the music." It was useless to cloak it, or to attempt to dodge the responsibility.

Dr. Beck, of Indiana, moved an indefinite postponement of the whole subject.

Other gentlemen rose to speak, but the president decided that a motion to postpone was not debatable.

Dr. Jewell rose to a point of order, and protested against being "gagged." [The president here reversed his decision.] Dr. Jewell said that the action of the day previous was regretted, and that gentlemen had acted hastily. Many, who at first sight voted to accept the apologies, now regretted having done so.

Dr. Hamm, of Philadelphia, explained the action of the Philadelphia County Medical Society, and began to read a remonstrance from it, which he desired to incorporate into his speech.

Dr. Biddle objected to the reading of this remonstrance, as a violation of plighted faith.

It was here moved and decided that the association go into "committee of the whole," and Dr. Edwards, of Ohio, was called to the chair.

The apology of Dr. Reese was again taken up, and discussed with spirit, although there was no manifestation of bad feeling on either side. At length he presented the following:

"The undersigned regrets that he certified to the professional qualifications for Blockley Hospital, Philadelphia, of an expelled member of this body, and hereby offers this apology for his departure from the ethical code."

This was received with loud applause, and, on motion of Dr. White, accepted as an ample and satisfactory apology.

Dr. Bryan submitted a similar apology, which was also accepted, and then the committee adjourned until to-day at nine o'clock a. m., evidently well pleased that this question was finally disposed of.

HOSPITALITIES.

At five o'clock p. m., the delegates went in omnibuses provided for their use to Georgetown College, by invitation of the faculty. After examining this fine institution, which commands a magnificent view, and visiting its fine library, museum, and apparatus room, the party were hospitably entertained; after which they returned to this city. In the evening, there were entertainments given them at the residence of Dr. Thomas Miller, 246 E street; Dr. W. P. Johnson, 466 Seventh street; and Dr. A. Y. P. Garnett, 465 Ninth street.

### THIRD DAY.

The president, Dr. Lindsley, called the association to order at half-past nine o'clock. The secretary not having his minutes in readiness, the reading of the minutes of the day previous was dispensed with.

Dr. Grant, of New York, asked leave to present a complaint against the New York Medical College, but upon information by Dr. Edwards that a committee on ethics would be recommended by the nominating committee, he withdrew his request.

The minutes were then read. Several proposals to amend them were made, and either ruled out of order or withdrawn.

The appointment during last year of Dr. Geo. Haywood, of Boston, as a delegate to represent the American Medical Association in kindred societies in Europe, was announced by Dr. Eve.

Dr. Hamilton, from the committee of delegates from medical schools and colleges, to whom was referred the report of the special committee on medical colleges, reported the following preamble and resolution:

Fully appreciating the value and importance of the resolution under which they were appointed, but a majority of the gentlemen constituting this committee not being authorized by the medical faculties of the several colleges with which we are connected to act as their representatives in this matter, and therefore regarding it quite impossible to secure a convention of delegates in the interim of the meetings of the association: therefore,

*Resolved*, That we recommend to all the medical colleges entitled to a representation in this body that they appoint delegates, especially instructed to represent them in a meeting to be held at Louisville on Monday, the day immediately preceding the convention of the American Medical Association, for the year 1859, at ten o'clock, at such place as the committee of arrangements shall designate.

The report was accepted, and the preamble and resolutions were passed; after which several gentlemen claimed the floor, but the president decided that the reports of special committees were in order, the reports of committees on medical topography and epidemics having previously been referred to the committee on publication without reading.

Dr. Foster Jenkins, of New York, read a report on the spontaneous umbilical hemorrhage of the newly born; which was read and referred to the committee on publication.

Dr. S. M. Bemis, of Kentucky, read an able and learned report on the "influence of marriages of consanguinity upon offspring."

Dr. Palmer, of Detroit, read a report, made by Dr. E. Andrews, of Chicago, Illinois, on the "functions of different portions of the cerebellum."

Dr. Campbell, of Georgia, read a report on the "nervous concomitants of febrile diseases," which was accepted and referred to the committee of publication.

Dr. J. Marion Simms, of New York city, read an abstract of his report on the treatment and of the results of obstructed labor, illustrated with a series of magnified illustrations.

Dr. Stephenson, of New York, read an interesting abstract of his report on "the treatment best adapted to each variety of cataract, with the method of operation, place of selection, time, age," etc.

On motion, other reports were called for, read by their titles, and referred to the committee of publication.

### COMMITTEES FOR THE ENSUING YEAR.

Dr. Edwards, from the committee of nomination, offered the following list of committees for the ensuing year, which was accepted, and the committees were chosen:

*Special Committee on the Microscope*—Drs. Holston of Ohio, Dalton of New York, Hutchinson of Indiana, Stout of California, and Ellis of Massachusetts.

*Special Committee on Medical Jurisprudence*—Drs. Smith of New York, Hamilton of Buffalo, Crosby of New Hampshire, Purple of New York, and Mulford of New Jersey.

*Committee on Quarantine*—Drs. Harris of New York, Moriarty of Massachusetts, La Roche of Pennsylvania, Wragg of South Carolina, and Fenner of New Orleans.

*Committee on Surgical Pathology*—Dr. James R. Wood, of New York, chairman.

*Committee on Diseases and Mortality of Boarding Schools*—Dr. C. P. Mallengly, of Kentucky, chairman.

*Committee on the various Surgical Operations for the Relief of Defective Vision*—Dr. Montrose A. Pallen, of St. Louis, chairman.

*Committee on Milk Sickness*—Dr. Edward A. Murphy, of Indiana, chairman.

*Committee on Medical Ethics*—Drs. John Watson of New York, Dalton of Massachusetts, Emerson of Pennsylvania, Hamilton of New York, and Gaillard of South Carolina.

Dr. Edwards also reported from the committee of nomination the following resolution:

*Resolved*, That a committee of nine be appointed by the chair to wait on the Hon. Howell Cobb, Secretary of the Treasury, and respectfully to request the restoration of Dr. M. J. Bailey as inspector of drugs and medicines for the port of New York.

Dr. Wilcox, of Connecticut, offered an amendment to the resolution of Dr. Edwards, "disclaiming all political considerations." The amendment was accepted by Dr. Edwards.

The resolution as amended was then carried by a vote of 79 ayes to 52 noes.

*Resolved*, That a committee of nine be appointed by the chair to wait on the Hon. Howell Cobb, Secretary of the Treasury, and respectfully to request the restoration of Dr. M. J. Bailey as inspector of drugs and medicines for the port of New York—at the same time disclaiming all political considerations.

Dr. Bohrer, of Georgetown, chairman of the committee on special medical essays, stated that they had not had time to read, much less consider, the papers placed in their hands.

On motion, the committee on special medical essays was instructed to hand such papers as they deemed worthy to the committee on publication.

The president announced, as a special committee to wait on the Secretary of the Treasury, Drs. Arnold of Georgia, Atkins of Virginia, Buckley of New York, Hayes of Pennsylvania, Smith of New Jersey, McPheeters of Missouri, Hargraves of Alabama, Pitcher of Michigan, and Hooker of Connecticut.

On motion, Dr. Edwards was added to the committee as chairman. He declined, giving personal reasons as an excuse, but the committee refused to receive it, and he was accordingly chosen.

A gentleman stated that he, with several friends, had voted for the resolution with the sole intention of moving its reconsideration.

Dr. Grant, of New Jersey, presented a complaint made by the Newark Medical Society against the New York Medical College, for a violation of the ethics of the profession. Dr. Edwards presented a similar complaint, and Dr. Oakley a complaint from the Union and Essex County Medical Societies. They were received and referred.

Dr. Sutton, of Kentucky, moved that Dr. Jarvis, of Massachusetts, have further time to report on a uniform system of registration of births, marriages, and deaths, and that a committee be appointed to urge upon the census bureau of 1860 the importance of having a physician attached to it to collect vital statistics.

Dr. Kyle, of Ohio, proposed an amendment to the constitution by which no person can sit as a member or a delegate at meetings of this association who is not a graduate of a recognized medical college. Laid over for one year, under the rules.

Dr. L. A. Smith presented resolutions of the New Jersey Medical Society, praying for such changes of the constitution as would establish a board of census in every judicial circuit of the Supreme Court, who should examine and grant diplomas to all proper members of this association. Laid over for one year, under the rules.

Dr. Humphries, of Indiana, presented a resolution praying for an interchange of transactions of state and county societies; which was adopted.

Dr. Boyle, chairman of the committee of arrangements, presented the names of Professor Swallow, of Missouri, and Professor Mittag, as "members by invitation," and they were elected.

An invitation from Professor Bache to visit the Coast Survey Bureaux, on Capitol Hill, was read, accepted, and a vote of thanks for the courtesy was passed.

Dr. Gibbs, of South Carolina, moved that Professor Henry be requested to favor the association with his views on meteorology at such time during the session as he may select; carried unanimously.

Dr. Campbell, of Georgia, moved that the secretary place on record an expression of the regret with which the society has learned the death of Drs. C. R. Walton, S. W. Granton, Marshall Hall, T. Y. Simmons, Mitchell, and other members deceased since last annual session; carried.

VOTE OF THANKS.

On motion of Dr. Phelps, the following resolutions were passed unanimously, the members rising:

*Resolved,* That the thanks of this association are eminently due to the Regents and Professor Henry, of the Smithsonian Institution, for the ample and convenient accommodation afforded for the transaction of business.

*Resolved,* That the committee of arrangements are entitled to our praise and highest appreciation of their exertions to promote the comfort of the members and the best interests of the association.

*Resolved,* That to the physicians of Washington and Georgetown and the faculty of Georgetown College we accord the homage of our sincerest thanks for their elegant hospitalities extended to the members from abroad, by which the pleasure of their sojourn here has been so greatly enhanced.

*Resolved,* That we feel assured that the impressions on the tablet of memory received here, in our national metropolis, in this the first year of the second decade of the association, will long remain an evidence of the urbane attentions received not only from the Chief Magistrate and other public functionaries of our glorious Union, but of private citizens and the community at large.

*Resolved*, That the manifestations of union of heart and purpose in the action of this session inaugurate a new era, and call for devout acknowledgment to Divine Providence, and presage, as we trust, not only a bright future for the association, but also as contributing to the perpetuity and prosperity of our great national confederation.

On motion of Dr. Anderson, of New Jersey, it was unanimously resolved that the thanks of the medical association be presented to Rev. Dr. McGuire and the faculty of the College of Georgetown for their very cordial reception and entertainment of the association at the College yesterday.

Dr. Arnold, of Georgia, then exhibited specimens of a new method of medical preparations of some membrane incomprehensible to the reporter, but which was evidently very interesting to the association.

Dr. Dunbar claimed the floor, and urged the reconsideration of the vote appointing a committee to wait on the Secretary of the Treasury, and solicit the reinstation of Dr. Bailey.

Dr. Payne, of Virginia, opposed the reconsideration.

Dr. Tyler advocated it, and asked if this association was formed to wait on executive officers, and to dictate to them whom they shall remove, and whom they shall appoint. Many gentlemen around him, he was assured, had voted for the resolution without due reflection, and he trusted with confidence in their sober second thought. [Applause.] The press and the profession, he felt confident, would denounce this association if it entered into the wide field of politics. It was instituted to promote the great cause of science, not to join issue with government. [Applause.]

Dr. Morgan also advocated a reconsideration. He was not a partisan. Although he resides in Washington, he has no personal acquaintance with the President or the Secretary of the Treasury, but he was confident that they would not have made the change without good reason, and it was not the mission of this association to criticise or to attempt to change their views.

Dr. Palmer, after stating how little regard he had for the opinions of the press, inquired as to the present incumbent of the office. Is he capable?

Dr. Watson, of New York, said that Dr. Bailey had had his circulars out since his "rotation," and the subject had been twice before the Academy of Medicine, who have ignored it.

Dr. Burns, of Brooklyn, said that he was not a politician, and that he was a personal friend of Dr. Bailey, but he hoped that the vote would be reconsidered.

A member from California related his experience there on a question as to the superintendent of a lunatic asylum. In his opinion the less the association had to do with politics, or with expressions of opinion on political appointments, the better. [Applause.]

Dr. McNulty, of New York, said that the question had been twice before the New York Academy of Medicine, and twice been voted down. The present incumbent, whom it is sought to oust, is a German by birth and education. He can read the invoices in whatever European language they may be sent, and he makes his own analyses, which it is reported the ex-inspector did not do.

After some "parliamentary" skirmishing, it was decided to reconsider by a vote of 51 ayes to 32 noes. And, on motion, the subject was then indefinitely postponed.

The association then took a recess of two hours, for dinner.

### EVENING AND CLOSING SESSION.

The association was called to order at five o'clock p. m., by Dr. Sutton, one of the vice presidents, who took the chair.

The amendments to the constitution, proposed at the annual meeting at Nashville, had been made the "special order." They were:

1st. Amend the third article of the constitution, in relation to meetings, by inserting after the words "first Tuesday in May," the words "or the first Tuesday in June;" and also inserting after the words "shall be determined," the words "with the time of meeting." 2d. In article 2, omit the words "medical colleges," and also the words "the faculty of every regularly constituted medical college, or chartered school of medicine, shall have the privilege of sending two delegates."

Each amendment was separately discussed, and each was lost by a large vote. An amendment proposed at Philadelphia in 1856 providing for the establishment of a permanent secretaryship was lost by a vote of 53 ayes to 84 noes.

On motion of Dr. Foster, of Tennessee, the secretary was directed to collect all the by-laws, and have them printed in the next volume.

An attempt was made to introduce a motion endorsing the acoustics and ventilation of the new Capitol extensions, but it was ridiculed by Dr. Sayre, and was withdrawn.

Various additional votes of thanks were passed, and, at ten minutes of seven, the association adjourned *sine die*.

### EXCURSION.

The members and delegates of the National Medical Association were entertained last evening at the residences of Hon. Stephen A. Douglas, corner of New Jersey avenue and I street,

Dr. Cornelius Boyle, 27 Four-and-a-half street, and Dr. J. F. May, 397 C street.

To-day there will be an excursion on the steamboat Thomas Collyer to Mount Vernon, Fort Washington, and the Pavilion, where a *planked shad entertainment* will be given to the association by the medical profession of the District. The Thomas Collyer will leave her pier at the foot of Seventh street punctually at 15 minutes before 10 o'clock a. m. Ladies accompanying delegates have been invited to join the party.

